# Impact of mixed silages of licorice aerial parts and whole plant corn on nutrient digestibility, rumen fermentation, and gastrointestinal microbiota in Simmental cattle

**DOI:** 10.3389/fvets.2025.1658831

**Published:** 2025-10-09

**Authors:** Haonan Liu, Limin Tang, Xingyu Xu, Qifeng Gao, Yuliang Sun, Wenhao Li, Dong Lu, Houjun Yu, Tao Jiang

**Affiliations:** ^1^College of Animal Science and Technology, Tarim,University, Alar, China; ^2^Animal Husbandy and Veterinany Station of the Third Division, Tumushuke, China; ^3^Key Laboratory of Tarim Animal Husbandry Science and Technology, Xinjiang Production and Construction Corps, Tarim University, Alar, China; ^4^Key Laboratory of Livestock and Forage Resources Utilization around Tarim, Ministry of Agriculture and Rural Affairs, Tarim University, Alar, China

**Keywords:** licorice–corn mixed silage, apparent nutrient digestibility, rumen fermentation parameters, gastrointestinal microbiota, Simmental cattle

## Abstract

**Introduction:**

Licorice aerial parts are widely cultivated in China but often underutilized due to nutrient loss during haymaking. Ensiling with whole-plant corn may enhance their nutritional value and utilization in ruminant diets.

**Methods:**

This study evaluated the effects of mixed silages containing aerial parts of licorice and whole-plant corn at inclusion levels of 0, 22, 28, and 34% on nutrient digestibility, rumen fermentation, and gastrointestinal microbiota in Simmental cattle. Forty-eight male Simmental cattle were randomly assigned to four groups and fed the experimental diets for 75 days. Apparent nutrient digestibility, rumen fermentation parameters, and microbial profiles in rumen fluid and feces were analyzed.

**Results:**

The 22% and 28% silage groups showed significantly higher digestibility of neutral detergent fiber (NDf) and acid detergent filber (ADF), along with increased concentrations of rumen acetate and total volatile fatty acids (TVFA), compared to the control and 34% groups. Rumen pH was significantly lower in these groups. Microbial diversity (Chao1 index increased inthe 34% group, while the 22% group exhibited a higher relative abundance of beneficial rumen bacteria such as Oscillospiraceae and NK4A214 group, with Oscillospiraceae negatively correlated with rumen pH. in feces, Firmicutes was enriched in the 28%group and identified as a key biomarker, Other beneficial taxa, including Christensenellaceae, Monoglobaceae and Ruminococcus, also increased with silage supplementation.

**Discussion/conclusion:**

These findings suggest that incorporating 28% licorice-corn mixed silage into the diet optimizes nutrient digestibility, enhances rumen fermentation, and improves gut microbial composition, thereby boosting feeding efficiency in fattening Simmental cattle.

## Introduction

The shortage of high-quality roughage supplies is a major constraint to the development of the cattle industry in many developing countries. Southern Xinjiang, China’s largest area of saline soil and desertification, is characterized by low rainfall and poor soil fertility. Due to these harsh environmental conditions, the availability of high-quality roughage in southern Xinjiang is extremely limited. A promising solution is the promotion of saline-tolerant forage crops. Licorice (*Glycyrrhiza uralensis Fisch*), a perennial plant belonging to the genus *Glycyrrhiza* in the legume family, is one of the most valuable plant resources adapted to arid and semi-arid conditions (1). It is primarily distributed in the dry regions of northeastern, northern, and northwestern China, as well as Mongolia, Central Asia, and Russia, with Xinjiang being one of the main production areas (2, 3). The aerial parts of licorice are a promising roughage resource, containing over 16% crude protein (CP) and rich in bioactive compounds such as flavonoids and glycosides, which exhibit antiviral, antioxidant, and immunomodulatory activities (4–6). These nutritional and functional properties have supported their successful incorporation into feed for sheep (7, 8), poultry (9), and fish (10), where they have significantly enhanced growth performance. However, their use as a roughage is limited by practical challenges: during haymaking, they are prone to substantial nutrient losses, and their low water-soluble carbohydrate (WSC) content hinders effective ensiling. Corn silage, known as the “king of feed” for ruminants due to its low crude fiber, high palatability, and rich soluble sugars, is typically used in whole-plant form. Co-ensiling licorice aerial parts with whole-plant corn or sweet sorghum has been shown to improve silage quality and compensate for the limitations of licorice alone (11), offering a viable strategy to enhance their utilization as a high-quality roughage source.

*In vitro* studies have shown that increasing the proportion of licorice aerial parts beyond 4.5% of dietary dry matter impairs fermentation characteristics and gas production, suggesting threshold effects for optimal digestibility (4). *In vivo* trials in sheep supplemented with licorice extract up to 4.5% of DM reported enhanced antioxidant status and immune function without adverse effects on growth or intake, while higher levels reduced rumen fermentation efficiency (5). Furthermore, glycyrrhizic acid, the primary active compound in licorice, has been associated with dose-dependent toxicity, including hypertension and electrolyte imbalance, at excessive intake levels (6). Based on these findings, the inclusion levels of 22, 28, and 34% were selected to represent moderate, effective, and upper-limit doses to evaluate both beneficial and potentially adverse effects. We hypothesized that appropriate inclusion levels of mixed silage containing licorice aerial parts and whole-plant corn would not only improve silage fermentation quality, but also enhance nutrient digestibility, optimize rumen fermentation parameters, and beneficially modulate the gastrointestinal microbiota in Simmental cattle. Therefore, mixed silage composed of licorice aerial parts and whole-plant corn was selected for investigation. Simmental cattle were used as experimental animals, and different inclusion levels of the mixed silage were during the late fattening stage to evaluate the effects on apparent nutrient digestibility, rumen fermentation parameters, and gastrointestinal microbiota. The findings from this study would provide valuable insights for the practical application of mixed silage in ruminant nutrition.

## Materials and methods

### Mixed silages production

Aerial parts of licorice and whole-plant corn were sourced from Tumshuk City, Xinjiang Production and Construction Corps, China. Samples were collected on September 21, 2023. The sampling location is situated between 78°07′–78°21′E, and 39°22′–39°30’N, at an elevation ranging from 1,100 m to 2,063 m, and features a temperate continental arid climate.

Both the licorice aerial parts and whole-plant corn were harvested using a silage harvester and chopped to a length of approximately 1–2 cm. The moisture content was adjusted to 65–70%, and the two components were mixed at a mass ratio of 1:1. The mixture was thoroughly blended, compacted into a silo, tightly sealed with a double-layer plastic film. The white outer layer reflected sunlight to reduce heat accumulation, while the black inner layer blocked light to inhibit the growth of aerobic bacteria. The edges of the film were secured using sandbags, tires, or soil to prevent air leakage and maintain anaerobic conditions. The silage was fermented at room temperature for 90 days before use. The main nutritional composition of the mixed silage is presented in Table 1.

**Table 1:** The main nutrient levels of mixed silage (dry matter basis, %).

Items	Aerial parts of licorice	Whole plant corn	Mixed silage
Dry matter (DM)	32.85	26.53	27.05
Crude protein (CP)	12.83	5.29	9.35
Ether Extract(EE)	5.63	2.54	4.89
Crude fibre(CF)	15.45	9.73	13.26
Neutral detergent fibre(NDF)	49.26	40.58	36.52
Acid detergent fibre(ADF)	36.23	24.24	23.64
Calcium (Ca)	2.14	0.24	1.34
Phosphorus (P)	0.86	0.03	0.11

### Experimental design

A completely randomized design was employed for this study. Forty-eight male Simmental cattle, approximately 18–20 months old, each weighing 550 ± 10 kg and with a body condition score (BCS) of 6, were randomly assigned to four groups (*n* = 12 per group). The groups received total mixed rations (TMR) supplemented with 0% (control), 22, 28%, or 34% mixed silage, respectively. The TMRs were formulated based on nutritional requirements outlined in the Chinese Feeding Standards for Beef Cattle (NY/ T815-2004) (7). The ingredient composition and nutritional values of each treatment group are provided in Table 2. Nutrient concentrations of crude protein (CP), calcium (Ca), and phosphorus (P) in the feed supplied was determined according to standard AOAC methods (8). CP was measured using the Kjeldahl method, Ca was determined viapotassium permanganate titration, and P content was measured by absorbance photometry (Table 2).

**Table 2:** Basic diet composition and nutritional level (dry matter basis, %).

Items	Groups			
	0% mixed silages	22% mixed silages	28% mixed silages	34% mixed silages
Composition				
Mixed silage		22	28	34
Corn silage	22	-	-	-
Wheat stalks	25.33	27.22	23.33	20
Corn	37	41	40.44	40.77
Flax meal	10.04	4.5	3	0
Salt	1	1	1	1
Stone powder	1.53	1.53	1.53	1.53
Baking soda	1.6	1.2	1.2	1.2
Premix^1^	1.5	1.5	1.5	1.5
Nutrient Levels				
Metabolic energy, MJ/kg	11.52	11.56	11.58	11.60
DMI, /kg/d	9.25	9.30	9.32	9.27
NDF %	33.21	33.32	33.63	33.04
ADF %	26.43	26.48	26.52	26.53
CP %	10.59	10.65	10.91	11.10
Ca %	0.33	0.33	0.40	0.44
P %	0.41	0.51	0.53	0.61

1 Each kilogram of basal diet contains: Copper, 10 mg; Manganese, 20 mg; Zinc, 30 mg; Iodine, 0.3 mg; Selenium, 0.25 mg; Cobalt, 0.25 mg; Vitamin D₃, 1,650 IU; Vitamin E, 120 IU; Ammonium chloride, 2,700 mg; Baking soda, 10,000 mg; Salt, 4,000 mg.

### Animal feeding and management

The experimental cattle were housed in tie stalls for a total of 75 days, which included a 15-day adaptation period and a 60-day experimental feeding period. All cattle were dewormed and ear-tagged prior to the start of the trial. Animals were fed twice daily, at 10 am and 6 pm, with feed offered ad libitum and free access to clean drinking water provided throughout the trial. To ensure proper feed intake, feed bunks were checked daily before the morning feeding, and refusals were maintained between 0 and 5% of the total feed offered.

Sample collection: 5 days before the end of the experiment, feed and residual materials were collected, thoroughly mixed, and quartered. Subsamples were oven-dried at 65 °C to a constant weight, ground, and stored in self-sealing bags for later nutrient analysis. On day 60, rumen fluid was collected from each anima using a rumen fluid collector. The first aliquot was discarded to avoid contamination, and subsequent fluid was collected. Rumen contents were filtered through four layers of sterile gauze. The pH of the filtrate was measured immediately and the remainder was stored in 5 mL cryovials at −80 °C for analysis of rumen fermentation parameters and microbial composition via 16S rDNA sequencing. Between days 56 and 60, 400 g of fresh feces were collected daily from the rectum of each animal. A portion of each sample was treated with 10% sulfuric acid to preserve nitrogen content. Fecal samples from each animal were pooled over the 5-day period, dried at 65 °C, ground, and stored for determination of apparent nutrient digestibility. Additional fecal samples were snap-frozen in liquid nitrogen and stored at −80 °C for subsequent microbial analysis via 16S rDNA sequencing.

### Analysis of nutritional content and apparent digestibility of feed

Feed and fecal samples were ground and passed through a 40-mesh sieve prior to analysis. Dry matter (DM) was determined by drying at 105 °C in a forced-air oven for 4 h. Nitrogen (N) content in feed, feces, urine and microbial sampleswere measured using the Kjeldahl method, and crude protein (CP) was calculated as N × 6.25 (8). Ash content was determined by complete combustion in a muffle furnace at 600 °C for 6 h (8). Neutral detergent fiber (NDF) and acid detergent fiber (ADF) were according to the Van Soest method (9). Acid-insoluble ash (AIA) in feces and feed served as an internal markerfor calculating the apparent digestibility of each nutrient.

Apparent digestibility (%) was calculated using the following equation; 100–100 x (AIA content in the diet/AIA in feces) × (nutrient in feces/ The amount of this nutrient in the diet) (10).

### Analysis of rumen fermentation

The pH of the rumen fluid was measured immediately after collection using a portable pH meter (FE28, Mettler Toledo, China). Ammonia nitrogen (NH₃-N) concentrations were measured using Broderick’s alkaline sodium hypochlorite-phenol spectrophotometry method (11). Volatile fatty acids (VFAs), including acetate, propionate, and butyrate, were quantified using gas chromatography (SP7800, Beijing Jingke Ruida, China) following the method of Yang et al. (12). The chromatographic conditions were as follows: capillary column GP-sil88 (30 m × 0.25 mm × 0.25 μm); flame ionization detector (FID) at 260 °C; inlet temperature 230 °C; injection volume 1 μL. Carrier gas pressures were: nitrogen 0.04 MPa, hydrogen 0.05 MPa, air 0.05 MPa, and tail blow (make-up gas) 0.05 MPa.

### Rumen liquid and fecal samples 16S rDNA gene analysis

#### DNA extraction PCR amplification, and product purification

Genomic DNA was extracted using the PowerSoil® DNA Isolation Kit (Qiagen, United States) according to the manufacturer’s instructions. The purity and concentration of the extracted DNA were assessed by 1% agarose gel electrophoresis. The 16S rDNA gene was amplified using the universal bacterial primers 27F (AGRGTTTGATYNTGGCTCAG) and 1492R (TASGGHTACCTTGTTASGACTT). PCR reactions were performed in a 20 μL reaction system, containing 1 μL of each forward and reverse primers, 20 μL of Solexa PCR mix, and 5–50 ng of template DNA. PCR was performed with an initial denaturation at 95 °C for 5 min, followed by 30 cycles of 95 °C for 30 s, 50 °C for 30 s, and 72 °C for 1 min/kb, with a final extension at 72 °C for 7 min. PCR products were quantified, and samples were pooled according to the required sequencing depth and fragment size for each sample. The pooled PCR products were purified using 0.8 × volume of magnetic beads to remove impurities and primer dimers.

#### Library preparation and sequencing

Sequencing libraries were generated using the NEB Next® Ultra™ II FS DNA PCR-free Library Prep Kit (New England Biolabs, United States, Catalog #: E7430L) following manufacturer’s recommendations, and unique indexes were added to each sample. The libraries were quantified usinga Qubit fluorometer and real-time PCR, and the fragment size distribution was assessed using a Bioanalyzer. Quantified libraries were pooled based on effective concentration and required data output, and sequencing was performed on the PacBio Sequel II platform.

#### Paired-end reads assembly and quality control

Raw subreads were processed to obtain circular consensus sequencing (CCS) reads using SMRT Link software (version 8.0). Demultiplexing of CCS reads was performed with the Lima tool (version 1.7.0) based on barcode sequences to separate samples. Chimeric sequences were identified and removed using UCHIME (version 8.1) (13), resulting in a set of high-quality, non-chimeric CCS reads for downstream analysis.

#### Operational taxonomic units denoise and taxonomic annotation

Sequences were clustered into operational taxonomic units (OTUs) at a 97% similarity threshold using USEARCH (version 10.0) (14), with a minimum abundance filter set at 0.005% of the total sequence count to remove low-frequency noise (15). Taxonomic annotation of representative OTU sequences was performed using the RDP Classifier (version 2.2)^1^ with a confidence threshold of 0.8. Classification was conducted against the SILVA 16S rRNA database^2^ using Clustal W/X (version 2.0) for alignment.

#### Alpha diversity, beta diversity and LEfSe analysis

Alpha diversity indices, including the Shannon and Chao1 indices, were calculated using Mothur software (version 1.3).^3^ Beta diversity was assessed using non-metric multidimensional scaling (NMDS) based on Bray-Curtis and unweighted UniFrac distances, visualized with the ggplot2 and ade4 packages in R (version 3.5.3). LEfSe analysis^4^ was conducted to identify significant bacterial biomarkers differentiating between groups.

### Statistical analysis

Differences in apparent nutrient digestibility and rumen fermentation parameters were analyzed by one-way ANOVA, followed by Duncan’s multiple range test for post-hoc comparisons. Microbial community analysis, including statistical comparisons of relative bacterial abundance, was performed by Tsingke Biotechnology Co., Ltd. (Beijing, China). The company conducted normality testing to determine the suitability of parametric methods and applied the Benjamini–Hochberg false discovery rate (FDR) correction to adjust *p*-values for multiple comparisons. Results were expressed as means ± SEM, and *p* < 0.05 was considered statistically significant. Graphs were generated using OriginPro 2021 (version 9.8.0.200).

## Results

### Effect of mixed silage of aerial parts of licorice with whole plant corn on apparent nutrient digestibility in Simmental cattle

The apparent nutrient digestibility of Simmental cattle fed diets containing different proportions of mixed silage composed of aerial parts of licorice and whole plant corn is presented in Table 3. As the proportion of mixed silage increased, the apparent digestibility of nutrients initially increased and then declined. Notably, the crude protein (CP) digestibility in the 34% mixed silage group was significantly lower compared to the 22 and 28% mixed silage groups (*p* = 0.017). The neutral detergent fiber (NDF) and acid detergent fiber (ADF) digestibility in the 22 and 28% groups were significantly higher than those in the control group (0%) and the 34% group (*p* = 0.001 for NDF; *p* = 0.009 for ADF) No significant differences were observed in the ether extract (EE) digestibility among all treatment groups (*p* > 0.05; Table 3).

**Table 3:** Effect of mixed silages of aerial parts of licorice with whole plant corn on apparent digestibility of nutrients in Simmental cattle.

Items	Groups				Mean ±SEM	*p*-value
	0% mixed silages	22% mixed silages	28% mixed silages	34% mixed silages		
CP digestibility %	57.87^ab^	72.26^a^	67.61^a^	46.28^b^	3.28	0.017
EE digestibility %	60.61	79.63	76.34	67.75	3.15	0.114
NDF digestibility %	47.23^b^	68.40^a^	69.94^a^	41.40^b^	4.06	0.001
ADF digestibility %	33.08^b^	58.42^a^	56.98^a^	30.31^b^	4.55	0.009

Different letters within a row indicate significant differences (*p* < 0.05).

### Effect of mixed silage of aerial parts of licorice with whole plant corn on rumen fermentation parameters in Simmental cattle

The rumen fermentation parameters of Simmental cattle fed mixed silage containing different proportions of aerial parts of licorice and whole plant corn are presented in Table 4. As the proportion of mixed silage increased the concentrations of ruminal acetate, propionate, and TVFA initially increased and then decreased. The 22 and 28% mixed silage groups showed significantly higher concentrations of acetate and TVFA compared to the control and 34% mixed silage groups (*p* = 0.001). Rumen pH was significantly higher in the control and 34% mixed silage groups than in the 22 and 28% mixed silage groups (*p* = 0.001). Propionate concentrations were also significantly higher in in the 22 and 28% groups compared to the 34% group (*p* = 0.042). No significant differences were observed in ruminal butyrate and NH₃-N concentrations among the groups (*p* > 0.05; Table 4).

**Table 4:** Effect of mixed silages of aerial parts of licorice with whole plant corn on rumen fermentation parameters in Simmental cattle.

Items	Groups				Mean ±SEM	*p*
	0% mixed silages	22% mixed silages	28% mixed silages	34% mixed silages		
Acetate, mmol/L	55.41^b^	66.12^a^	65.97^a^	56.40^b^	1.70	0.003
Propionate, mmol/L	12.43^ab^	14.70^a^	14.53^a^	12.00^b^	0.46	0.042
Butyrate, mmol/L	6.42	7.60	6.95	7.11	0.20	0.221
TVFA, mmol/L	74.26^b^	88.42^a^	87.46^a^	75.50^b^	2.12	0.001
pH	6.54^a^	6.27^b^	6.30^b^	6.48^a^	0.04	0.001
NH_3_-N, mg/100 mL	9.23	8.92	9.88	9.88	0.29	0.630

Different letters within a row indicate significant differences (*p* < 0.05).

### Effect of mixed silages of aerial parts of licorice with whole plant corn on gastrointestinal microbiome in Simmental cattle

#### Effect of mixed silages of aerial parts of licorice with whole plant corn on bacterial species richness and diversity in Simmental cattle

**Figure 1:**
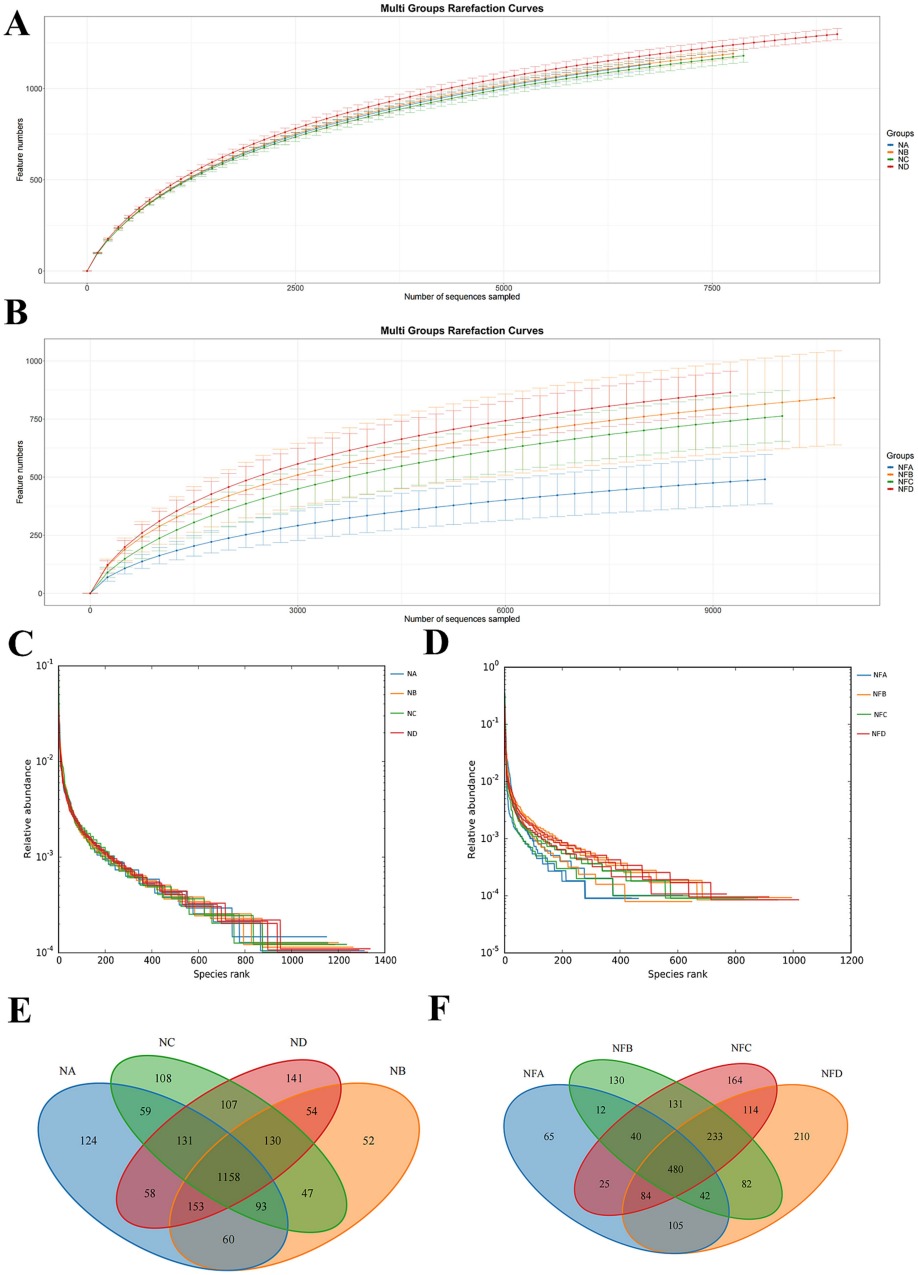
Microbial community diversity and structural analysis in Simmental cattle. **(A,B)** Rarefaction curves illustrating sequencing depth and observed amplicon sequence variant (ASV) richness for rumen **(A)** and fecal **(B)** samples. A plateau indicates sufficient sequencing depth to capture the majority of microbial diversity within a sample. **(C,D)** Hierarchical clustering diagrams (dendrograms) based on microbial community dissimilarity, showing relationships among rumen **(C)** and fecal **(D)** samples. Closer branches indicate more similar microbial community compositions. **(E,F)** Venn diagrams of shared and unique amplicon sequence variants (ASVs) among different treatment groups in rumen **(E)** and fecal **(F)** samples, illustrating community overlap and distinct ASV profiles. Sample groups are defined as: NA (0% mixed silage rumen), NB (22% mixed silage rumen), NC (28% mixed silage rumen), ND (another 22% mixed silage rumen); NFA (0% mixed silage fecal), NFB (22% mixed silage fecal), NFC (28% mixed silage fecal), NFD (another 22% mixed silage fecal).

The rarefaction curves based on the number of Features indicate that when the original sequence number exceeds 7,500, the number of detected species plateaus, suggesting that the sequencing depth was sufficient to cover the majority of microbial diversity (Figures 1A,B). Hierarchical clustering analysis further showed a relatively homogeneous distribution of microbial species among samples (Figures 1C,D). Operational Taxonomic Units (OTUs) were clustered from rumen and fecal samples. According to the Venn diagrams, the rumen samples showed 124 unique OTUs in the control group, 52 in the 22% mixed silage group, 108 in the 28% group, and 141 in the 34% group, with 488 shared core OTUs (Figure 1E). In fecal samples, the control group had 65 unique OTUs, while the 22, 28, and 34% mixed silage groups had 130, 164, and 210 unique OTUs, respectively. The number of shared core OTUs in feces was also 488 (Figure 1F).

#### Effect of mixed silages of aerial parts of licorice with whole plant corn on bacterial effective sequence, alpha diversity and Beta diversity in Simmental cattle

**Figure 2:**
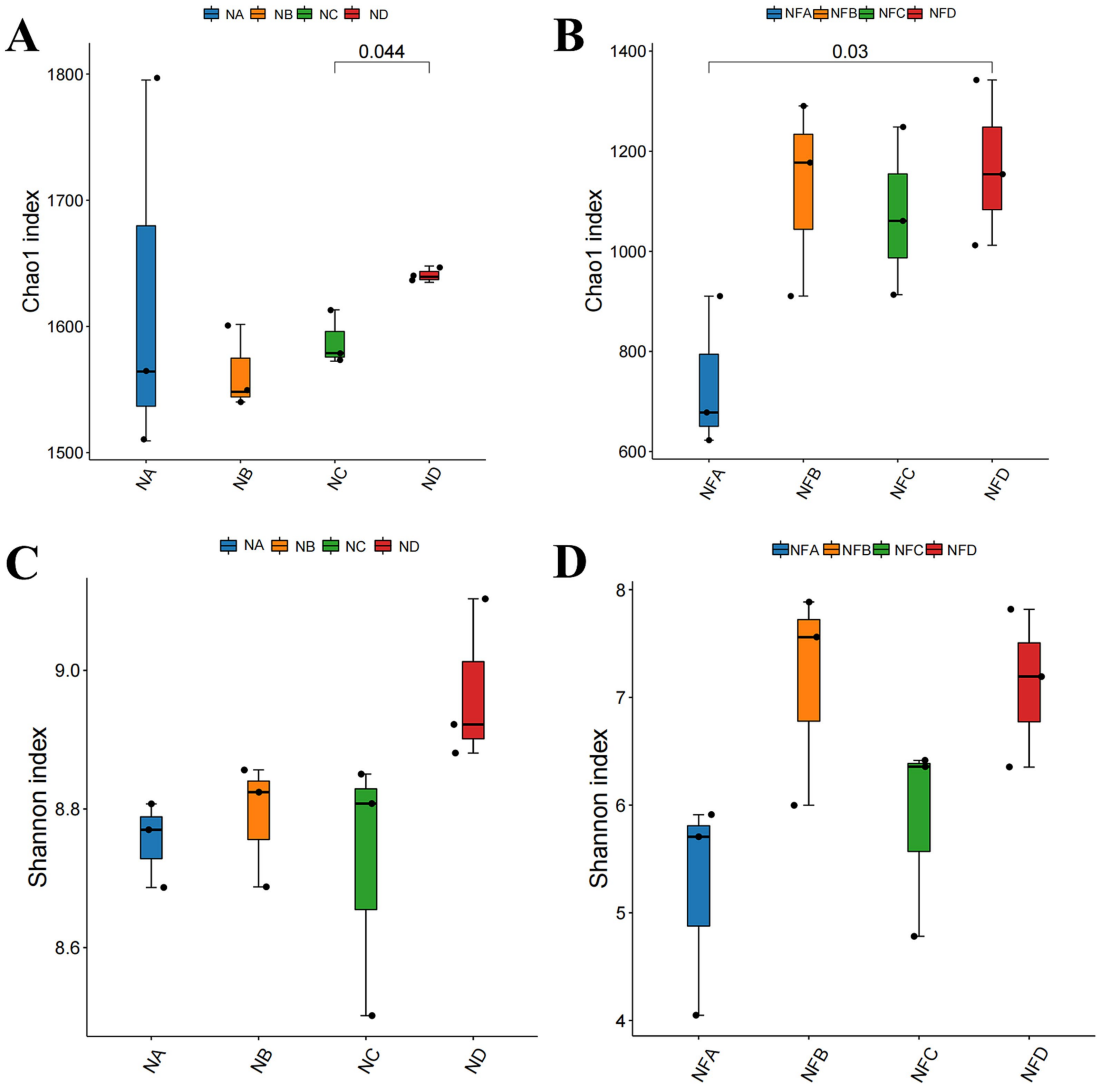
Alpha diversity indices of gastrointestinal microbiota in different treatment groups. Alpha diversity represents the diversity within a single sample. **(A)** Rumen microbial alpha diversity (e.g., Chao1 index); **(B)** Rumen microbial alpha diversity (e.g., Shannon index); **(C)** Fecal microbial alpha diversity (e.g., Chao1 index); **(D)** Fecal microbial alpha diversity (e.g., Shannon index). Different lowercase letters above bars indicate statistically significant differences (*p* < 0.05) among groups. Sample groups are defined as: NA, NB, NC, ND for rumen; NFA, NFB, NFC, NFD for fecal.

After splicing and quality filtering of 16S rDNA sequencing data from rumen and fecal samples, a total of 289,500 high-quality Circular Consensus Sequences (CCS) were obtained, averaging 12,062.5 sequences per sample. Clustering at 100% similarity produced 24,000 operational taxonomic units (OTUs). Alpha diversity analysis showed significant differences in the rumen Chao1 index between the 28 and 34% mixed silage groups (*p* = 0.044), as well as in the fecal Chao1 index between the control and 34% mixed silage groups (*p* = 0.03; Figures 2A,B). Although there were no statistically significant differences in the Shannon index for either rumen or fecal microbiota (*p* > 0.05), the trends in Shannon index were consistent with those of the Chao1 index (Figures 2C,D).

**Figure 3:**
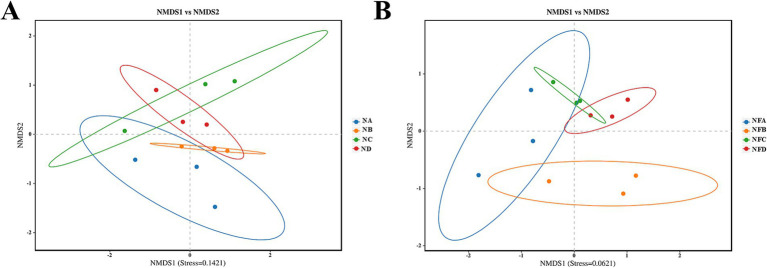
Non-metric Multidimensional Scaling (NMDS) plots illustrating beta diversity of gastrointestinal microbial communities in different treatment groups. Beta diversity represents the dissimilarity in microbial community composition between samples. **(A)** Rumen microbial beta diversity; **(B)** Fecal microbial beta diversity. Each point represents a sample, and the distance between points reflects the dissimilarity of their microbial communities. Sample sizes for each group are *n* = 3. Sample groups are defined as: NA, NB, NC, ND for rumen; NFA, NFB, NFC, NFD for fecal.

Beta diversity analysis using Bray-Curtis distance-based non-metric multidimensional scaling (NMDS) showed stress values below 0.2, indicating reliable ordination. Notably, supplementation with different levels of mixed silage resulted in distinct shifts in both rumen and fecal microbial communities (Figure 3).

#### Effect of mixed silages of aerial parts of licorice with whole plant corn on bacterial species composition in Simmental cattle

##### Effect of mixed silages of aerial parts of licorice with whole plant corn on the composition of rumen bacteria in Simmental cattle

**Figure 4:**
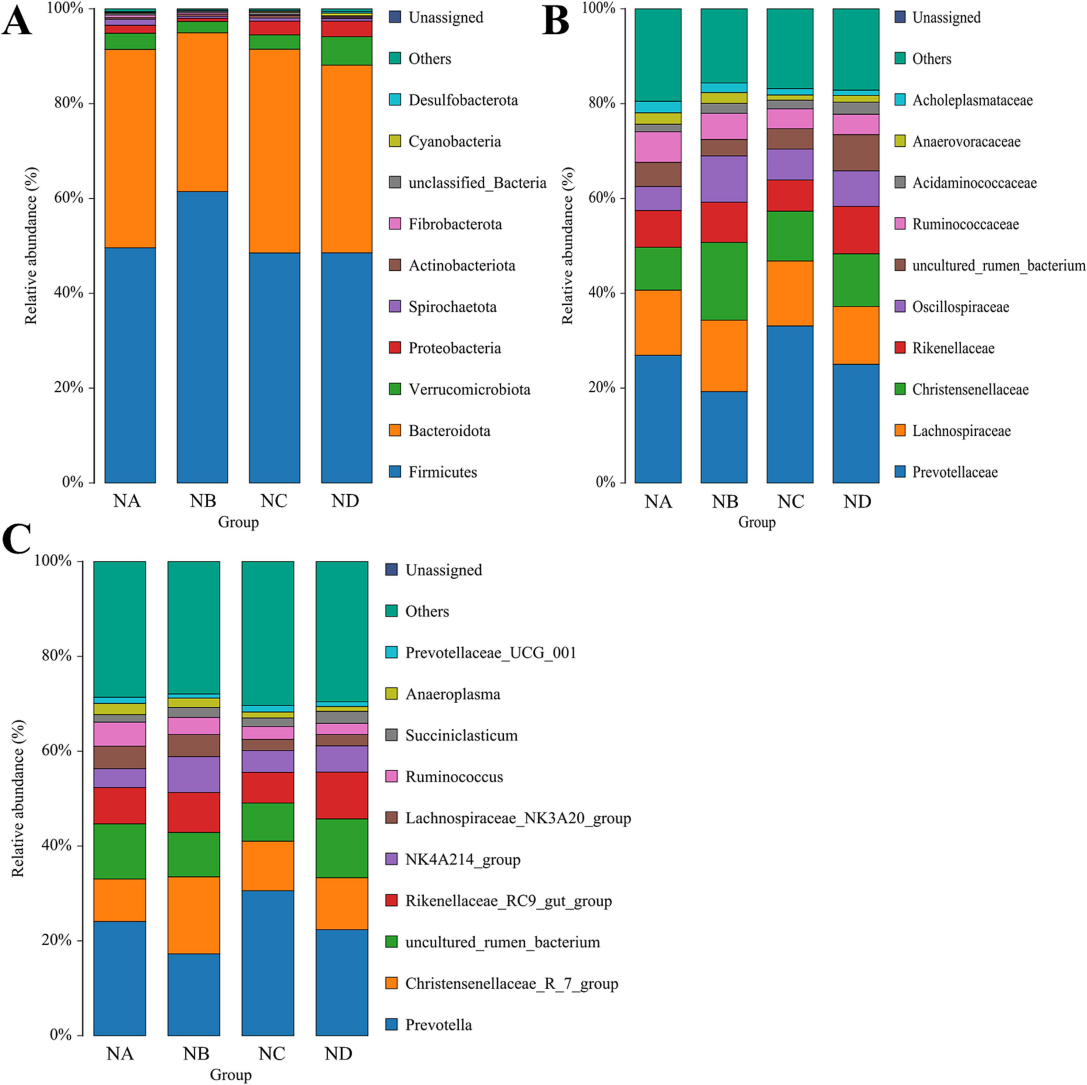
Bar charts showing the relative abundances of rumen bacteria in different treatment groups. **(A)** Phylum level; **(B)** Family level; **(C)** Genus level. Only taxa with relative abundance greater than 1% are displayed to highlight dominant microbial groups. These hierarchical levels illustrate the overall structure and key taxonomic shifts within the rumen microbial community. Sample groups are defined as: NA, NB, NC, ND.

A total of 23 phyla, 167 families, and 329 genera were identified in the rumen samples. *Bacteroidota* and *Firmicutes* were the dominant phyla across all groups, together accounting for over 80% of the total relative abundance, with no significant differences observed among groups (*p* > 0.05; Figure 4A). The relative abundance of *Desulfobacterota* was significantly higher in the 34% mixed silage group compared to the control group (*p* = 0.009). *Prevotellaceae* and *Lachnospiraceae* were the dominant families in the rumen across all groups, with *Lachnospiraceae* having a relative abundance greater than 10%. In addition, the relative abundance of *Oscillospiraceae* in the 22% mixed silage group was significantly greater than that in the control group (*p* = 0.037; Figure 4B). *Prevotella* was the predominant genus in the rumen samples of all groups, with an average relative abundance exceeding 20%. The relative abundance of *NK4A214_group* in the 22% mixed silage group was significantly higher than in the control group (*p* = 0.035; Figure 4C).

##### Effect of mixed silages of aerial parts of licorice with whole plant corn on the composition of fecal bacteria in Simmental cattle

**Figure 5:**
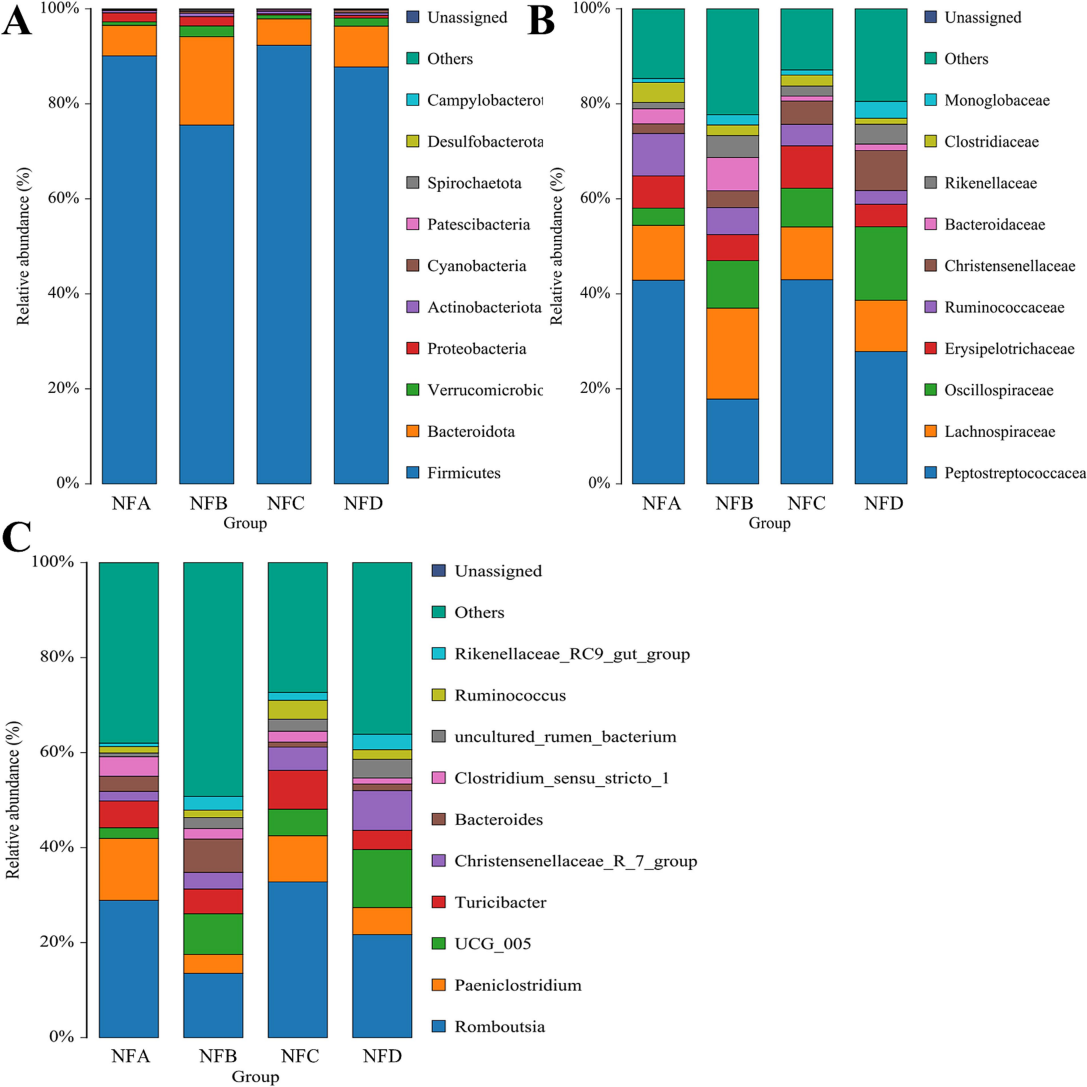
Bar charts showing the relative abundances of fecal bacteria in different treatment groups. **(A)** Phylum level; **(B)** Family level; **(C)** Genus level. Only taxa with relative abundance greater than 1% are displayed to highlight dominant microbial groups. These hierarchical levels illustrate the overall structure and key taxonomic shifts within the fecal microbial community. Sample groups are defined as: NFA, NFB, NFC, NFD.

A total of 18 phyla, 153 families, and 327 genera were identified in fecal samples. *Firmicutes* and *Bacteroidetes* were the predominant fecal bacterial phyla across all groups, together accounting for more than 90% of the total relative abundance, with *Firmicutes* showing an absolute predominance. The relative abundance of *Firmicutes* was significantly higher in the 28% mixed silage group compared to the 22% mixed silage group (*p* = 0.038), while *Bacteroidetes* were more abundant in the 22% mixed silage group compared to the control and 28% mixed silage groups (*p* = 0.046). In addition, the relative abundance of *Cyanobacteria* in the 34% mixed silage group was significantly higher than in the control and 22% mixed silage groups (*p* = 0.007; Figure 5A).

At the family level, *Peptostreptococcaceae* and *Lachnospiraceae* were dominant in fecal samples, with *Peptostreptococcaceae* accounting for over 30% of the total relative abundance. *Lachnospiraceae* also exhibited a relative abundance exceeding 10%. The 34% mixed silage group showed a significantly higher relative abundance of *Oscillospiraceae* compared to the control group (*p* = 0.031). *Christensenellaceae* were significantly more abundant in all mixed silage groups (22, 28, and 34%) compared to the control group (*p* < 0.001). Conversely, *Clostridiaceae* were more abundant in the control group than in the 34% mixed silage group (*p* = 0.039). Additionally, *Monoglobaceae* were significantly more abundant in the 34% mixed silage group compared to the control and 28% mixed silage groups (Figure 5B).

At the genus level, *Romboutsia* was the dominant genus across all groups, with an average relative abundance above 20%. The relative abundance of *UCG_005* was significantly higher in the 34% mixed silage group compared to the control group (*p* = 0.027). *Christensenellaceae_R-7_group* showed a markedly higher relative abundance in the 34% mixed silage group compared to the control, 22, and 28% mixed silage groups (*p* < 0.0001). In contrast, *Clostridium_sensu_stricto_1* was significantly more abundant in the control group than in the 28 and 34% mixed silage groups (*p* = 0.042). *Ruminococcus* was significantly more abundant in the 28% mixed silage group compared to the control group (*p* = 0.030). Finally, *Rikenellaceae_RC9_gut_group* was significantly more abundant in the 34% mixed silage group than in the control group (*p* = 0.022; Figure 5C).

#### LEfSe analysis of Simmental cattle bacteria in mixed silages

##### LEfSe analysis of Simmental cattle rumen bacteria in mixed silages

**Figure 6:**
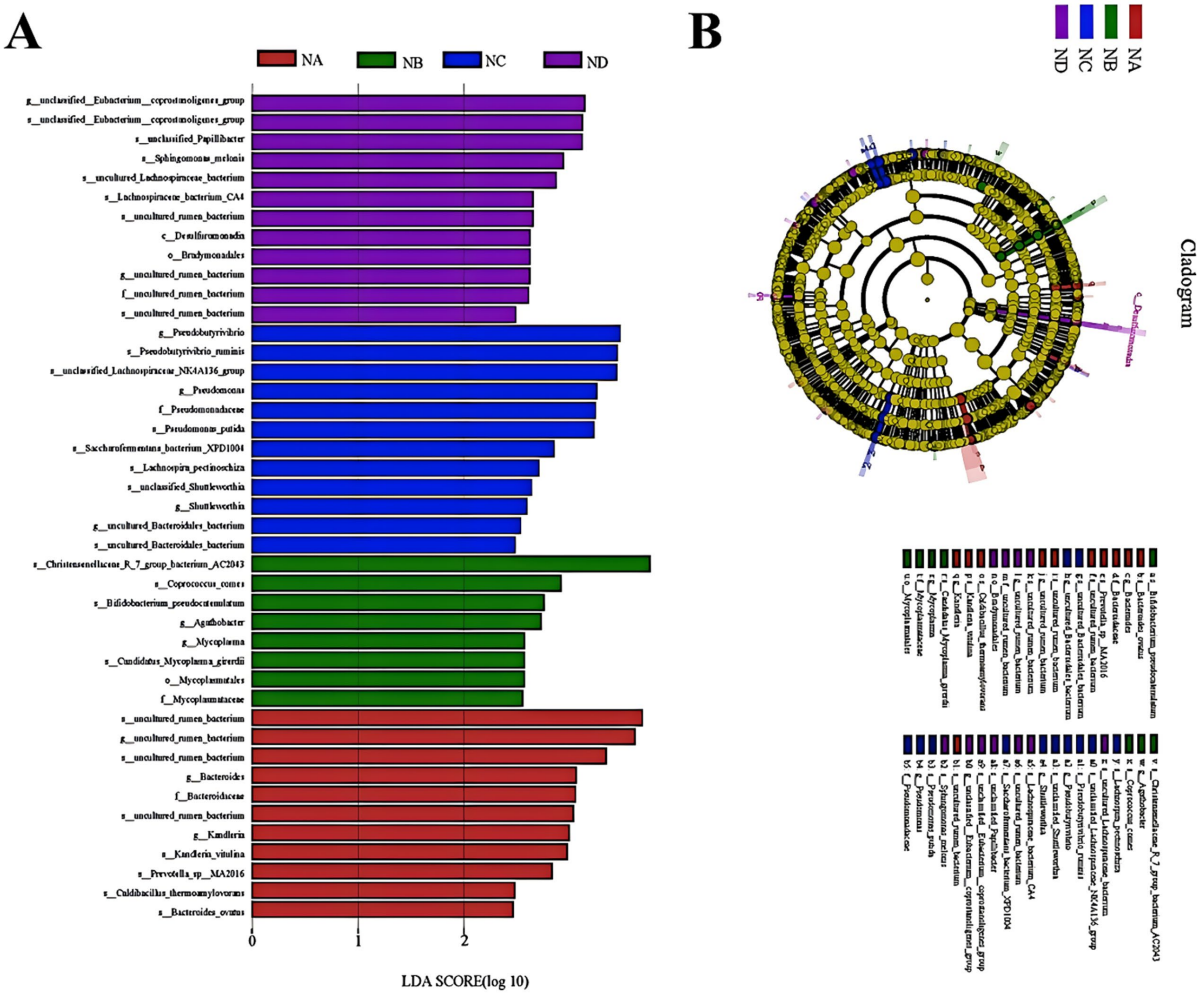
Linear Discriminant Analysis Effect Size (LEfSe) analysis identifying differentially abundant rumen bacterial taxa with an LDA score greater than 2.0. **(A)** LDA score distribution plot showing the effect size of each differentially abundant taxon. The length of the bar indicates the LDA score (log 10), representing the magnitude of the difference in abundance. Colors correspond to the group in which the taxon is enriched: NA (red), NB (green), NC (blue), ND (purple). **(B)** Taxonomic cladogram illustrating the phylogenetic relationships of the significantly enriched taxa identified by LEfSe analysis. The concentric circles represent taxonomic levels from the outermost to the innermost: phylum, class, order, family, genus, and species. Colored nodes and shading in the phylogenetic tree represent taxa that are significantly enriched in the corresponding group, while yellow nodes indicate taxa with no significant differences among groups. This cladogram visually represents the taxonomic hierarchy and enrichment patterns of the taxa shown in the LDA score plot **(A)**. Sample groups are defined as: NA, NB, NC, ND.

To further investigate the key bacterial taxa influenced by mixed silage in the rumen of Simmental cattle, LEfSe analysis was performed to identify significant biomarkers. The analysis revealed 11, 8, 12, and 12 key biomarkers in the control group, 22% mixed silage group, 28% mixed silage group, and 34% mixed silage group, respectively (Figure 6A). These biomarkers were detected across various taxonomic levels, including phylum, family, and genus. However, some bacteria at these taxonomic levels were not identified as significant biomarkers (Figure 6B).

#### LEfSe analysis of Simmental cattle fecal bacteria in mixed silages

**Figure 7:**
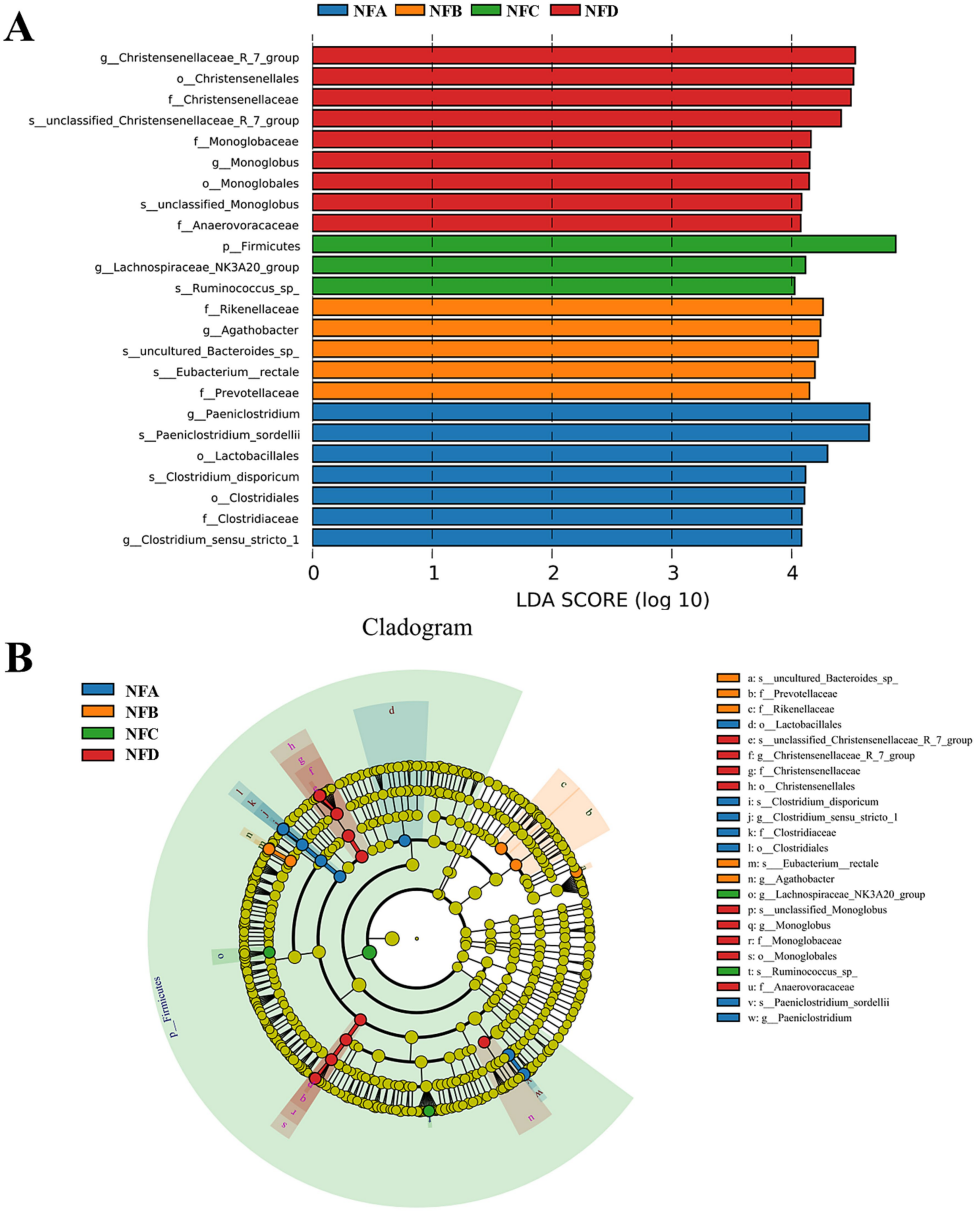
Linear Discriminant Analysis Effect Size (LEfSe) analysis identifying differentially abundant fecal bacterial taxa with an LDA score greater than 4.0. **(A)** LDA score distribution plot showing the effect size of each differentially abundant taxon. The length of the bar indicates the LDA score (log 10), representing the magnitude of the difference in abundance. Colors correspond to the group in which the taxon is enriched: NFA (red), NFB (green), NFC (blue), NFD (purple). The higher LDA score threshold (4.0) highlights more robust or highly abundant biomarkers. **(B)** Taxonomic cladogram illustrating the phylogenetic relationships of the significantly enriched taxa identified by LEfSe analysis. The concentric circles represent taxonomic levels from the outermost to the innermost: phylum, class, order, family, genus, and species. Colored nodes and shading in the phylogenetic tree represent bacterial taxa significantly enriched in the corresponding group, while yellow nodes indicate taxa with no significant differences among groups. This cladogram visually represents the taxonomic hierarchy and enrichment patterns of the taxa shown in the LDA score plot **(A)**. Sample groups are defined as: NFA, NFB, NFC, NFD.

To further investigate the key bacterial taxa influenced by mixed silage in the feces of Simmental cattle, LEfSe analysis was performed. The analysis revealed 7, 5, 3, and 9 taxa with differential abundance (LDA > 2) in the control, 22, 28, and 34% mixed silage groups, respectively (Figure 7A). While Firmicutes were enriched in the 28% mixed silage group, this reflects a broader trend in microbial composition rather than a precise biomarker. Within this phylum, genera such as Ruminococcus and families like Christensenellaceae and Lachnospiraceae, which are associated with fiber degradation and gut health, contributed substantially to the LEfSe signal. In contrast, Clostridiaceae was enriched in the control group, and Christensenellaceae, Monoglobaceae, and Christensenellaceae_R-7_group were more abundant in the 34% group (Figure 7B).

### Correlation analysis between gastrointestinal bacteria, apparent digestibility of nutrients, and rumen fermentation

**Figure 8:**
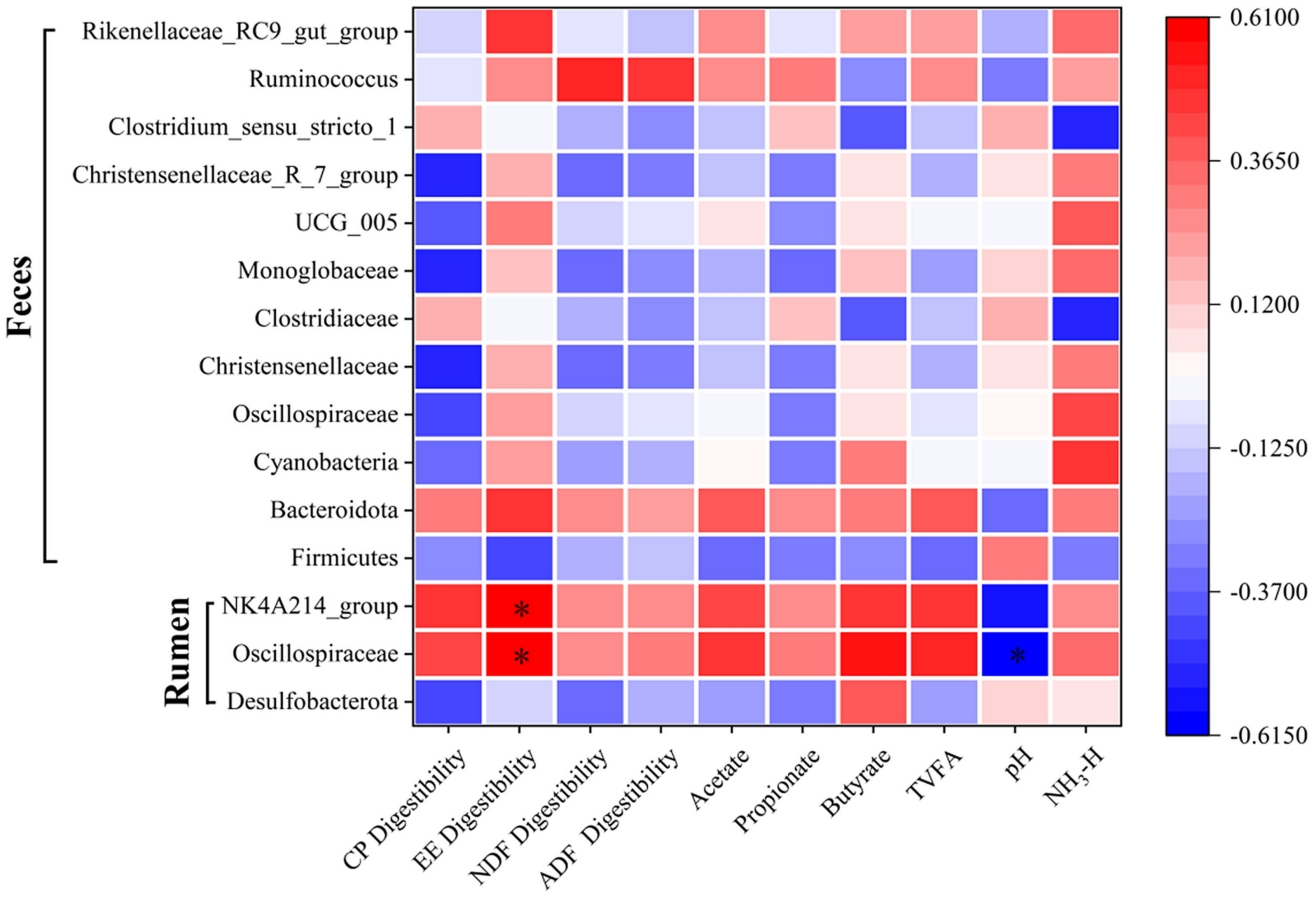
Spearman correlation analysis between gastrointestinal bacterial taxa, apparent nutrient digestibility parameters, and rumen fermentation parameters. The heatmap visualizes the correlation coefficients, where color intensity and direction (e.g., blue for positive, red for negative) indicate the strength and nature of the relationship. Key bacterial taxa (e.g., [List a few examples of specific taxa or taxonomic levels included]), apparent nutrient digestibility parameters (e.g., dry matter digestibility, crude protein digestibility, neutral detergent fiber digestibility), and rumen fermentation parameters (e.g., pH, total volatile fatty acids, acetate, propionate, butyrate) were included in the analysis. An asterisk (*) indicates a statistically significant correlation (*p* < 0.05).

An analysis of the associations among rumen and fecal microbial differences, apparent nutrient digestibility, and rumen fermentation parameters was conducted. The abundance of rumen *Oscillospiraceae* showed a significant positive correlation with ether extract (EE) digestibility and a significant negative correlation with rumen pH (*p* < 0.0001). Similarly, the rumen *NK4A214_group* was negatively correlated with rumen pH (*p* < 0.0001; Figure 8).

## Discussion

### Effect of mixed silages of aerial parts of licorice with whole plant corn on apparent digestibility of nutrients in Simmental cattle

Apparent nutrient digestibility reflects the digestion and absorption efficiency of nutrients from feed and serves as a key indicator for evaluating feed nutritional value (16). Guo et al. (5) reported that supplementing with licorice extract can improve apparent nutrient digestibility in Karakul sheep. Similarly, Chen et al. (4) found that an appropriate proportion of mixed silage with forage grass (sweet sorghum) and aerial parts of licorice mixed silage significantly increased the degradationrate of CP, ADF, and NDF in the sheep rumen. In this study, as the proportion of mixed silage increased, apparent nutrient digestibility first increased and then decreased. Specifically, apparent digestibility of NDF and ADF significantly improved in the 22 and 28% mixed silage groups, with CP digestibility peaking at the 28% mixed silage group. Previous research suggested that licorice stems and leaves might inhibit fiber-degrading bacteria, and when their proportion in the diet is too high, rumen fermentation can be suppressed (17). This may explain the reduced digestibility of CP, NDF, and ADF observed in the 34% mixed silage group. This reduction at higher licorice inclusion aligns with earlier findings that excessive levels of licorice aerial parts or glycyrrhizic acid can suppress microbial fermentation or alter ruminal balance, especially when exceeding ~4.5% of dietary dry matter. Overall, these results indicate that mixed silages of aerial parts of licorice with whole plant corn can enhance the apparent digestibility of CP, ADF, and NDF in Simmental cattle, with an optimal inclusion rate not exceeding 28%. Effect of mixed silages of aerial parts of licorice with whole plant corn on rumen fermentation parameters in Simmental cattle.

Rumen pH reflects the coordinated acid–base balance maintained by the rumen microbial population and host metabolism (18), and it typically shows a negative correlation with VFA concentrations (19). In this study, as the proportion of mixed silage increased, the contents of rumen acetate and total volatile fatty acids (TVFA) showed an opposite trend to rumen pH. Specifically, the highest concentrations of acetate and TVFA were observed in the 22 and 28% mixed silage groups, while the lowest were found in the control (0%) and 34% groups. This pattern aligns with the improved fiber digestibility seen at moderate inclusion levels. A prior study has reported that licorice extract supplementation reduced TVFA and acetate levels in the rumen of Karakul sheep while increasing propionate and butyrate concentrations (5). Moreover, *in vitro* experiments showed no significant effect of licorice extract on VFA synthesis (20). In the present study, propionate levels aligned with these prior findings, whereas acetate and TVFA did not. Propionate is mainly produced by the fermentation of starch and soluble sugars, while acetate primarily derives from the fermentation of fiber and semi-fiber components (16). Considering the apparent nutrient digestibility data from this study, NDF and ADF digestibility significantly increased in the 22 and 28% mixed silage groups, which likely contributed to the elevated acetate and TVFA concentrations observed.

Rumen ammonia nitrogen (NH3-N) content serves as an indicator of microbial protein metabolism from dietary nitrogen (21), and there is generally a negative relationship between protein digestibility and ammonia nitrogen levels. For example, replacing legume fodder with oat hay has been shown to improve nitrogen utilization efficiency in Simmental cattle (22). In this study, rumen NH3-N content did not differ significantly across mixed silage groups, despite the increased crude protein digestibility in the 22 and 28% groups. This may indicate that the mixed silages of aerial parts of licorice with whole plant corn facilitated enhanced protein degradation and utilization. However, as NH₃-N levels remained unchanged, the interpretation of efficient nitrogen capture remains speculative in the absence of direct measures such as microbial nitrogen incorporation or blood urea concentrations. Further investigation is needed to clarify the nitrogen utilization pathways involved.

### Effect of mixed silages of aerial parts of licorice with whole plant corn on bacterial alpha diversity and Beta diversity in Simmental cattle

Intestinal bacteria play a crucial role in animal digestion, nutrient absorption, and immune regulation. In ruminants, rumen microorganisms are particularly important for fiber degradation, fermentation of sugars and starch, and microbial protein synthesis (23). The structure and composition of the gut microbiota are influenced by several factors, including diet composition and feeding strategy. Alpha diversity reflects the richness and evenness of microbial communities. The Chao1 index indicates microbial abundance, while the Shannon index reflects community diversity (24). In this study, the rumen Chao1 index was significantly higher in the 34% mixed silage group compared to the 28% group. Similarly, in fecal samples, the Chao1 index of the 34% mixed silage group was significantly greater than that of the control group. Although the Shannon index followed a similar trend in both rumen and feces, the differences were not statistically significant. These findings may be partially influenced by the bioactive components of licorice stems and leaves, which are rich in flavonoids such as liquiritin, isoliquiritigenin, and glabridin (25). Some studies suggest that specific flavonoids can promote microbial richness and diversity by modulating gut microbial composition and supporting beneficial bacteria (26). However, as flavonoid effects can vary depending on structure, dosage, and microbial context, further research is needed to confirm their specific role in this setting. NMDS (non-metric multidimensional scaling) analysis based on Bray-Curtis distance further confirmed that different proportions of mixed silage led to distinct alterations in both rumen and fecal microbial communities. Overall, these results suggest that supplementing diets with mixed silages of aerial parts of licorice and whole plant corn may influence the structure and enhance the diversity of gastrointestinal microbiota in Simmental cattle; however, further studies are needed to determine whether these microbial shifts translate into improved gut function or feed efficiency. Effect of mixed silages of aerial parts of licorice with whole plant corn on bacterial species composition in Simmental cattle.

The rumen microbiome is a complex and dynamic ecosystem essential for nutrient digestion and absorption in ruminants (27). Previous studies have shown that *Bacteroidetes* and *Firmicutes* are the dominant bacterial phyla in Simmental cattle (28, 29). Consistent with these findings, *Bacteroidetes* and *Firmicutes* were the most abundant phyla in both rumen and feces in this study. *Bacteroidetes* are primarily involved in the fermentation of carbohydrates and polysaccharides in the plant cell wall (30), whereas *Firmicutes* contribute significantly to the degradation of cellulose, proteins, and other carbohydrates (31). In this study, there were no significant differences in the relative abundance of *Bacteroidetes* in the rumen across groups. However, in fecal samples, the 22% mixed silage group showed a higher relative abundance of *Bacteroidetes*, whereas *Firmicutes* were more abundant in the 28% group, where they were also identified as biomarkers. Considering the nutrient digestibility and volatile fatty acid (VFA) profiles, it appears that supplementation with mixed silages of aerial parts of licorice and whole plant corn may enhance the relative abundance of both *Bacteroidetes* and *Firmicutes*, thereby improving nutrient digestion and utilization. At the family level, *Prevotellaceae*, *Lachnospiraceae*, and *Peptostreptococcaceae* were dominant in both rumen and feces, which is consistent with the microbiota typically found in beef cattle (32). Notably, the 22% mixed silage group exhibited a significantly higher abundance of *Oscillospiraceae* in the rumen, while the 34% mixed silage group had increased levels of *Oscillospiraceae*, *Christensenellaceae*, and *Monoglobaceae*. *Oscillospiraceae* are associated with carbohydrate digestion and the production of short-chain fatty acids (33). *Christensenellaceae* are involved in protein catabolism and gut metabolite regulation (34), and are thought to support gut health and possibly alleviate inflammatory bowel conditions (35, 36). *Monoglobaceae* are strongly linked with host immunoinflammation (37) and have been noted as biomarker taxa in migratory yaks (38). Considering the enhancements in neutral detergent fiber (NDF), acid detergent fiber (ADF), and crude protein (CP) digestibility, along with increased total VFAs (TVFA), and the observed negative correlation between *Oscillospiraceae* and rumen pH, these results support the idea that dietary inclusion of licorice-based silages can beneficially modulate gastrointestinal microbiota. This modulation leads to enhanced nutrient utilization, in part due to the established inverse relationship between rumen pH and VFA concentration (19).

At the genus level, the relative abundance of *NK4A214_group* in the rumen was significantly increased in the mixed silage groups. Moreover, higher relative abundances of *Ruminococcus*, *UCG_005*, *Christensenellaceae_R_7_group*, and *Rikenellaceae_RC9_gut_group* were observed, while *Clostridium_sensu_stricto_1* was significantly reduced. *Rikenellaceae_RC9_gut_group* plays a role in degrading both soluble polysaccharides and insoluble cellulose, producing succinate and propionate, and alleviating intestinal inflammation (39, 40). *Ruminococcus* is a key fibrolytic genus that efficiently digests hemicellulose and cellulose (41). *NK4A214_group* is associated with acetate production and plays a role in host energy metabolism (42). In this study, increased digestibility of NDF, ADF, CP, and higher TVFA levels were observed alongside these microbial changes. Taken together, the results indicate that mixed silages of aerial parts of licorice with whole plant corn can enhance the abundance of beneficial microbial taxa such as *Rikenellaceae_RC9_gut_group*, *Christensenellaceae*, and *Ruminococcus*. This improvement supports better carbohydrate metabolism, energy availability, and immune function in Simmental cattle.

## Conclusion

Feeding Simmental cattle with a diet containing 28% mixed silages of aerial parts of licorice and whole plant corn significantly improved the apparent digestibility of NDF and ADF, and increased rumen acetate and total volatile fatty acid (TVFA) concentrations. This dietary treatment also enhanced gastrointestinal microbial diversity, with notable increases in the relative abundance of *Oscillospiraceae* and *NK4A214_group* in the rumen. In the feces, members of the phylum *Firmicutes* were enriched in the 28% mixed silage group, reflecting a broader microbial trend. More specifically, LEfSe analysis indicated that this enrichment was driven by genera such as *Ruminococcus* and families including *Christensenellaceae* and *Lachnospiraceae*, which are associated with fiber degradation and gut health. Additionally, the relative abundances of *Oscillospiraceae*, *Christensenellaceae*, *Monoglobaceae*, *UCG_005*, *Christensenellaceae_R_7_group*, *Ruminococcus*, and *Rikenellaceae_RC9_gut_group* were significantly elevated in the feces.

Based on these findings, we recommend that the inclusion of licorice aerial parts in mixed silage be limited to no more than 28% of the total dry matter, as this level provided the most favorable effects on nutrient digestibility, fermentation characteristics, antioxidant capacity, and microbial balance without adverse outcomes.

## Data Availability

The datasets presented in this study can be found in online repositories. The names of the repository/repositories and accession number(s) can be found at: https://www.ncbi.nlm.nih.gov/, 1148123.

## Footnotes

^1^ http://sourceforge.net/projects/rdpclassifier/

^2^ http://www.arb-silva.de

^3^ http://www.mothur.org/

^4^ http://huttenhower.sph.harvard.edu/lefse/
